# *In vitro* assessment of *Dracocephalum lindbergii* for growth inhibition, apoptosis induction, and cytokine modulation against *Leishmaniamajor*

**DOI:** 10.1016/j.heliyon.2024.e38331

**Published:** 2024-09-24

**Authors:** Faham Khamesipour, Ali Khamesipour, Seyed Hossein Hejazi, Mustafa Ghanadian

**Affiliations:** a Center for Research and Training in Skin Diseases and Leprosy, Tehran University of Medical Sciences, Tehran, Iran; b Department of Parasitology and Mycology, School of Medicine, Isfahan University of Medical Sciences, Isfahan, Iran; c Department of Pharmacognosy, School of Pharmacy and Pharmaceutical Sciences, Pharmaceutical Sciences Research Center, Isfahan University of Medical Sciences, Isfahan, Iran

**Keywords:** *Dracocephalum lindbergii*, Flowcytometry, Apoptosis, Traditional medicine, *Leishmania major*

## Abstract

Leishmaniasis is a vector-borne disease caused by several species of flagellate protozoan parasites belonging to the genus *Leishmania*, which are part of the Trypanosomatidae family. This study aims to evaluate the *in vitro* anti-*Leishmania* activity of *Dracocephalum lindbergii* Rech.f (*D. lindbergii*) on the growth, apoptosis induction, and proliferation of *Leishmania major*. To conduct this study, the aerial parts of *D. lindbergii* were collected during the flowering stage in May 2022 from North Khorasan province, Iran. The plant material was extracted using various solvents, starting with those of lower polarity and progressing to those of higher polarity. To assess the impact of the *D. lindbergii* fraction on *L. major* promastigotes, amastigotes, and macrophages (THP-1), cell viability was determined using the MTT assay. Additionally, flow cytometry with the Annexin V-PE apoptosis detection kit was employed to distinguish between viable, necrotic, and apoptotic promastigotes in response to treatment with the 100 % methanolic fraction of *D. lindbergii* (D). The expression levels of TNF-α, IFN-γ, IL-12, IL-10, TGF-β, IL4, iNOS, and GAPDH were quantified using real-time PCR (qPCR) on the macrophage cell line. Each treatment approach exhibited marked anti-leishmanial effects across different concentrations over 24, 48, and 72 h of incubation, showing statistically significant differences compared to the untreated control group (P < 0.001). Different concentrations of D, MAT, and AmpB, both individually and in combination, significantly reduced the total number of intramacrophage amastigotes compared to the untreated control groups at 24, 48, and 72 h. The results also showed time-dependent variations in the anti-*Leishmania* activity of the fraction. In terms of cellular morphology, treated cells exhibited changes such as shrinkage, cytoplasmic condensation, and reduced mobility, particularly noticeable after 24 h of treatment. Additionally, fraction D demonstrated significant antioxidant properties. This study highlights the potential of *D. lindbergii* as an anti-*Leishmania* agent, with the 100 % methanolic fraction emerging as a promising candidate for the development of novel treatments for leishmaniasis.

## 1 Introduction

Leishmaniasis, one of the most significant vector-borne human diseases, encompasses a group of illnesses caused by various species of flagellate protozoan parasites within the genus *Leishmania*, belonging to the Trypanosomatidae family. Currently, at least 20 species of *Leishmania* are recognized as infecting humans, with transmission occurring through female sandflies [1,2]. The disease presents with a diverse range of clinical symptoms, including skin lesions, and can affect mucous membranes, leading to disfiguring lesions in the nasal region. Some *Leishmania* species can damage internal organs, resulting in visceral leishmaniasis, a potentially fatal condition [3,4].

Leishmaniasis affects approximately 14 million individuals worldwide, with an annual incidence ranging from 0.9 to 1.6 million cases. It causes 20,000 to 30,000 fatalities each year, and around 350 million people are at risk of infection. This disease is ranked among the top six parasitic diseases in terms of priority, just below malaria [5,6]. Current treatment for leishmaniasis relies on pentavalent antimonials, drugs that have been in use for over half a century [2,5,6]. However, these substances are not only harmful but also face increasing resistance from the parasites [7].

Novel compounds specifically designed to combat parasitic illnesses have been identified through various screening tests. Extracts derived from plants, as well as pure compounds such as terpenoids and flavonoids (including quercetin and rotenone), have demonstrated significant anti-parasitic effects. Plants and natural products continue to be valuable resources in drug research due to their diverse chemical structures and promising long-term safety profiles [8,9]. The use of medicinal plants for treating diseases has a long history. Although chemically synthesized medicines dominate contemporary treatments, it is estimated that approximately one-third of all medicinal agents either originate from plants or have been adapted following extraction from plants sources [10].

*Dracocephalum lindbergii* Rech.f (*D. lindbergii*), a member of the Lamiaceae family, has a long history of traditional medicinal use for treating various disorders. Phytochemical studies have identified several primary constituents in the plant, including lignans, phytosterols, flavonoids, phenols, alkaloids, sesquiterpenes, and both oxygenated and hydrocarbon monoterpenes. These key components contribute to the plant's diverse effects, which include antihyperlipidemic, antimicrobial, anticancer, antispasmodic, antioxidant, and cardiovascular properties [11,12].

According to literature reports, bioactive components often play a crucial role in mediating the biological actions of plants. For instance, flavonoids are renowned for their antibacterial properties, while compounds such as limonene and α-terpineol contribute to the antinociceptive effects of plants [12]. Methoxylated flavones, including apigenin, luteolin, isokaempferid, crisimaritin, penduletin, and xanthomicrol, have been identified as responsible for anticancer properties [13]. Additionally, studies highlight the role of apigenin, a component found in certain medicinal plants, in inducing cellular apoptosis. Apoptosis is a regulated mechanism of cell death characterized by a sequence of energy-dependent processes and distinct morphological features [14,15].

Apigenin has demonstrated efficacy in inducing apoptosis through both the extrinsic pathway (via death receptors) and the intrinsic pathway (involving mitochondria) in human cancer cells. In prostate cancer cell lines PC-3 and DU145, apigenin treatment led to apoptosis, as evidenced by a dose-dependent reduction in *Bcl-2* and *Bcl-xL* protein levels and an increase in the active form of the *Bax* protein. Additionally, apigenin influenced the expression of mitochondrial proteins, elevating Bim levels and decreasing Mcl-1 expression. This interaction synergized with the *Bcl-2* inhibitor ABT-263, ultimately triggering apoptosis through mitochondrial pathways in the cells [16]. Moreover, phenolic compounds such as caffeic acid, chlorogenic acid, phenylpropanoids, and flavonoids are sometimes attributed with contributing to the antioxidant activity of medicinal plants [13].

Despite numerous studies on the biological properties of *D. lindbergii*, none have evaluated its *in vitro* efficacy on the growth and apoptosis induction in *L. major* (MRHO/IR/75/ER). It has been established that drugs capable of inducing apoptosis in *Leishmania* hold significant potential for effectively combating this parasitic disease [17]. *Leishmania major* (*L. major*) serve as a convenient screening model due to their robust proliferation in a cell-free environment [18]. The assessment of *L. major* inhibition occurs after approximately three days, during which the control organism undergoes 3- to 6-fold multiplication. Some researchers prefer this screening method for its simplicity, even though *L. major* are not the primary target parasites [18]. In this context, the current study was initiated with the objective of investigating the *in vitro* anti-*Leishmania* activity of *D. lindbergii* on the growth, apoptosis induction, and proliferation of *L major*.

## 2 Materials and methods

### 2.1 Plant material and solvent extraction

In May 2022, during the flowering, aerial parts of *D. lindbergii* were collected from North Khorasan Province, Iran, with permission from the land manager and authenticated by Dr. Seyed Mustafa Ghanadian at the Samsam-Shariat Herbarium, Isfahan University of Medical Sciences. The plant was positively identified and compared with a voucher specimen (SAM No. 4050).

Approximately 2500 g of the plant's leaves were air-dried, pulverized, and stored in a dark amber glass container. For extraction, 200 g of the powdered plant material were subjected to maceration with dichloromethane (F-1), methanol (F-2), and hexane (F-3), in that order, with continuous shaking for 48 h. The extracts were filtered, concentrated using a rotary evaporator, and then lyophilized [19]. The resulting powders were stored at −20 °C. Additionally, a 100 % methanolic fraction of *D. lindbergii* was prepared using 1500 g of plant material.

### 2.2 Phytochemical screening, identification of bioactive constituents, and NMR spectral analysis

The fractionation procedure utilized chromatography with a polyamide SC6 column (20 × 300 mm) and gradient solvent mixtures. Key fractions were selectively collected and subsequently recycled through an HPLC system. This recycling process utilized a Shimpak-RP C18 column (dimensions: 250 × 20 mm) with an acetonitrile: DMSO:water mobile phase (ratio of 69:9:22) to isolate and produce the main bioactive compounds. For further analysis, 1H NMR spectra of the isolated bioactive compounds were obtained using a standard 5 mm diameter probe. These spectra were acquired with a Bruker 400 MHz instrument (Bruker Biospin, Rheinstetten, Germany), operating at 400 MHz for ^1^H NMR spectroscopy and at 100 MHz for ^13^C NMR spectroscopy.

### 2.3 Isolation of bioactive compounds from the most potent extract (100 % methanolic fraction of *D. lindbergii*/D)

Based on the *in vitro* anti-leishmanial and cytotoxic activities of fractions, the 100 % methanolic fraction (D) from *D. lindbergii*, which exhibited stronger anti-*Leishmania* activity and the best an IC50 value, was selected for further purification. This fraction was separated using a SC6 polyamide column (20 × 300 mm) with gradient mixtures of chloroform and methanol in the following ratios: D.1 (100:0), D.2 (96:4), D.3 (94:6), D.4 (90:10), D.5 (88:12), D.6 (86:14), and D.7 (80:20). The major fractions D.2 and D.4 were then selected and injected into a Waters 600 HPLC pump with UV–vis detector at 250 nm (Waters Corporation, Milford, MA, USA), employing an end capped preparative Shimpak RP-C18 column, 20 × 250 mm, 5 μm (Shimadzu - Kyoto, Japan) with acetonitrile: dimethyl sulfoxide: water (in a ratio of 69:9:22) as the mobile phase with flowrate of 5 mL/min at room temperature for 60 min. This process yielded compounds 1 to 3 as the primary bioactive compounds.

### 2.4 NMR spectroscopy

**Fig. 1:**
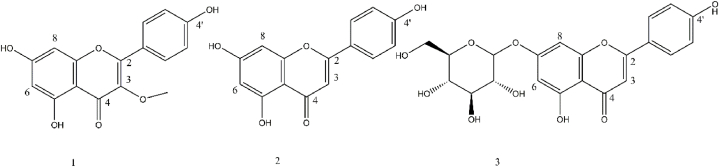
Identification of phytochemicals from the most active extract (100 % methanolic fraction from of *D. lindbergii*/D). Compounds were isolated by repeated chromatographic methods using a polyamide column and employing a Shimpak-RP C18 column (dimensions: 250 × 20 mm) with acetonitrile: DMSO: water (in a ratio of 69:9:22).

The 1H^1H1H NMR spectra of the isolated bioactive compounds were obtained using a 5 mm probe on a Bruker 400 MHz instrument (Bruker Biospin, Rheinstetten, Germany), operating at 400 MHz for 1H^1H1H NMR and 100 MHz for 13C^{13}C13C NMR. Spectra were recorded at 25 °C in DMSO-d6. Sugar linkages were identified through Heteronuclear Multiple Bond Correlation (HMBC) between the anomeric proton (H-1″) and its position on the aglycone. Anomeric proton configurations were determined to be in the β-form based on large coupling constants (J = 7.0–8.0 Hz). Chemical shifts, expressed in delta (*δ*_H_ or *δ*_C_), were calibrated after shimming using the topshim command in TOPSPIN software. Data analysis was performed using Mestrelab Research software (2018). The isolated compounds (Fig. 1) were characterized based on the NMR data and comparisons with published references.

Kaempferol 3-O-methyl ether [1]: ^1^H NMR in DMSO-d6, *δ*_H_ 12.6 (1H, s, OH),10.5 (1H, bs, OH), 7.97 (2H, d, J = 8.4 Hz, H-2′,6′), 6.92 (2H, d, *J* = 8.4 Hz, H-3′,5’), 6.38 (1H, d, *J* = 2.0 Hz, H-8), 6.21 (1H, d, *J* = 2.0 Hz, H-6) and 3.76 (3H, s, MeO). Negative ESI mass: 299 *m/z* [20].

Apigenin [2]: ^1^H NMR in DMSO-d6, *δ*_H_: 12.97 (1H, s, OH), 10.55 (1H, bs, OH), 7.93 (2H, d, *J* = 8.8 Hz, H-6′,2′), 6.93 (1H, d, *J* = 8.8 Hz, H-5′,3′), 6.79 (1H, s, H-3), 6.48 (1H, d, *J* = 2.0 Hz, H-8) and 6.19 (1H, d, *J* = 2.0 Hz, H-6); *δ*_C_: 183.2 (C4), 166.4 (C2), 163.8 (C7), 163.3 (C5), 163.2 (C4'), 159.0 (C9), 129.4 (C5', C3'), 117.3 (C6', C2'), 122.8 (C1'), 106.5 (C10), 104.4 (C3), 100.5 (C6), 95.3 (C8), Negative ESI mass: 269 *m/z* [19].

Apigenin-7-O-β-D-glucopyranoside [3]: ^1^H NMR in DMSO-d6, *δ*_H_ 12.9 (1H, s, 5-OH), 7.96 (2H, d, J = 8.4 Hz, H-2′,6′), 6.91 (2H, d, *J* = 8.4 Hz, H-3′,5’), 6.85 (1H, s, H-3), 6.77 (1H, d, *J* = 2.0 Hz, H-8), 6.46 (1H, d, *J* = 2.0 Hz, H-6), 5.12 (1H, d, *J* = 7.1 Hz, H-1″), 4.5 to 3.0 (6H, overlapped, H-1″ to H-6″). *δ*_C_: 182.02 (C4), 164.28 (C2), 162.97 (C5), 161.41 (C7), 161.13 (C9), 156.97 (C4'), 128.65 (C6', C2'), 121.02 (C1'), 116.02 (C5', C3'), 105.35 (C10), 103.12 (C3), 99.89 (C1″), 99.53, (C6), 94.87 (C8), 77.18 (C5″), 76.41 (C3″), 73.10 (C2″), 69.53 (C4″), 60.59 (C6″) Negative ESI mass: 431 *m/z* [19].

### 2.5 Parasites and cells

The promastigote form of *L. major* (MRHO/IR/75/ER), the national standard isolate, was obtained from the Pasteur Institute of Iran and cultured at 25 °C in RPMI-1640 medium containing 10 % heat-inactivated FBS and 1 % penicillin/streptomycin. The THP-1 macrophage cell line, provided by the Kerman Leishmaniasis Research Center, was grown in Dulbecco's Modified Eagle Medium (DMEM) supplemented with 10 % FBS and 0.5 % antibiotics (Sigma, Poole, UK), and incubated at 37 °C with 5 % CO₂ [21].

### 2.6 Cytotoxic effects

To assess the leishmanicidal effect on *L. major* intramacrophage amastigotes, macrophages (10⁷ cells) were cultured for 24 h. Subsequently, 200 μL of metacyclic promastigotes were introduced to the macrophages at a 10:1 ratio. After 24 h, 40 μL of different concentrations of D, MAT, AmpB, and their combinations were applied to the cells, followed by incubation for 24, 48, and 72 h. The cells were washed with 50 μL PBS to eliminate free parasites, fixed with methanol, and stained using Giemsa. The IC₅₀, defined as the concentration required to inhibit 50 % of the leishmanial organisms, was determined by counting Leishman bodies (amastigotes) in 100 macrophages under an optical microscope. All experiments were performed in triplicate [21].

### 2.7 MTT assay

To assess the impact of the *D. lindbergii* fraction on *L. major* promastigotes, amastigotes, and macrophages, cell viability was determined using the 3-(4,5-dimethylthiazol-2-yl)-2,5-diphenyl tetrazolium bromide (MTT). *L. major* in the logarithmic growth phase (1 × 10⁶) was plated in a 96-well microplate using RPMI-1640 medium supplemented with 10 % FBS and treated with different concentrations of the samples. The plates were then incubated. Subsequently, 10 μL of MTT (Sigma Chemical Co., St. Louis, MO) at a concentration of 5 mg/mL was added to each well. After incubation, DMSO was added to stop the reactions, and the optical density (OD) absorbance of the samples was measured at 570 nm using an ELISA plate reader (BioTek Company, USA). The results were analyzed by calculating the inhibition percentage through linear regression.

### 2.8 Determination of anti-promastigote activity (IC_50_)

Drug susceptibility assays were conducted using promastigotes in the logarithmic growth phase. A volume of 90 μL of cultured *L. major* promastigotes (1 × 10⁶ cells/mL) was transferred to each well of a 96-well microplate. Subsequently, 10 μL of the test compounds—D, meglumine antimoniate (MAT), Amphotericin B (Amp B), and their combinations (D + AmpB, D + MAT)—were added to the respective wells and incubated at 26 °C for 24, 48, and 72 h. The reactions were stopped, ELISA readings were taken, and the IC₅₀ (50 % inhibitory concentration) was calculated according to established protocols [22].

### 2.9 Anti-amastigote activity

To evaluate the leishmanicidal effects on *L. major* intramacrophage amastigotes, macrophages (1 × 10⁷ cells) were cultured for 24 h. Following this, 200 μL of metacyclic promastigotes were added to the macrophages at a 10:1 ratio. After another 24 h, 40 μL of various concentrations of D, meglumine antimoniate (MAT), Amphotericin B (Amp B), and their combinations were introduced to the cells and incubated for 24, 48, and 72 h. The cells were then washed with 50 μL PBS to remove any free parasites, fixed in methanol, and stained with Giemsa. The IC₅₀, or the concentration that inhibits 50 % of the leishmanial organisms, was assessed by counting Leishman bodies (amastigotes) in 100 macrophages under an optical microscope. Each experiment was conducted in triplicate, including negative controls (macrophages and parasites without treatment) and positive controls (macrophages and parasites treated with a known effective drug) for comparison.

### 2.10 Assessment of the antioxidant activity of D

The scavenging effect on 2,2-diphenyl-1-picrylhydrazyl (DPPH) free radicals was determined based on the reduction of DPPH as a stable free radical. The optical densities were measured at a wavelength of 517 nm. The elimination of free radicals was calculated using the following formula:

= (absorbance of control-absorbance of the sample) / (absorbance of control) × 100.

### 2.11 Th1 and Th2 related cytokines

The expression levels of selected Th1 and Th2 cytokines were quantified using quantitative real-time PCR (qRT-PCR) on infected macrophages. Total RNA was extracted from the samples using the High Pure RNA Isolation Kit (Roche, Basel, Switzerland). RNA concentration was measured with a Thermo Fisher Scientific NanoDrop, and cDNA was synthesized using the Roche Synthesis Kit. The qRT-PCR was performed following established protocols. Table 1 includes the primer sequences and details of the control genes. The experimental procedures were based on previous *in vitro* studies. Cycle threshold (CT) values were calculated using the formula: [ΔCT = CT (target) - CT (reference)], and fold change was determined using the comparative threshold method (2-ΔΔCT) [23].

**Table 1:** The specific primers and reference gene sequences.

Template	Forward and reverse sequences (5´-3´)		Product size (bp)
TNF-α	Forward	CAGGCGGTGCCTATGTCTC	161
	Reverse	CGATCACCCCGAAGTTCAGTAG	
IFN-γ	Forward	5-GCCGATGATCTCTCTCAAGTGAT-3	106
	Reverse	5-ACAGCAAGGCGAAAAAGGATG-3	
IL-12	Forward	TGGTTTGCCATCGTTTTGCTG	171
	Reverse	ACAGGTGAGGTTCACTGTTTCT	
IL-10	Forward	CTTACTGACTGGCATGAGGATCA	134
	Reverse	GCAGCTCTAGGAGCATGTGC	
TGF-β	Forward	CCACCTGCAAGACCATCGAC	112
	Reverse	CTGGCGAGCCTTAGTTTGGAC	
IL4	Forward	GGTCTCAACCCCCAGCTAGT	101
	Reverse	GCCGATGATCTCTCTCAAGTGAT	
iNOS	Forward	ACATCGACCCGTCCACAGTAT	89
	Reverse	CAGAGGGGTAGGCTTGTCTC	
	Reverse	GTGTGCCATCTCGTCAGTGAA	
GAPDH	Forward	5-AGGTCGGTGTGAACGGATTTG-3	95
	Reverse	5-GGGGTCGTTGATGGCAACA-3	

### 2.12 Apoptotic profile with IC_50_ concentration of D

Apoptosis in L. *major* amastigotes was evaluated using flow cytometry with the BD Annexin V/PE Apoptosis Detection Kit (Fisher Scientific). Amastigotes (10³ cells per well) were cultured in twelve-well plates and treated with IC₅₀ concentrations of D, meglumine antimoniate (MAT), Amphotericin B (AmpB), or their combinations for 72 h. After treatment, the parasites were washed with PBS and incubated in the dark at 25 °C with 5 μL of PE-Annexin V and 7 μL of 7-AAD for 15 min. The proportion of apoptotic parasites was then evaluated using FlowJo software.

### 2.13 Statistical analysis

Data were analyzed using SPSS software (Version 20.0, Chicago, IL, USA) and GraphPad Prism (Version 8.0). Two-way ANOVA was used to analyze cell viability data, and t-tests were employed to assess statistical significance where appropriate.

## 3 Results

### 3.1 Antileishmanial activity against *L. major* promastigotes

**Fig. 2:**
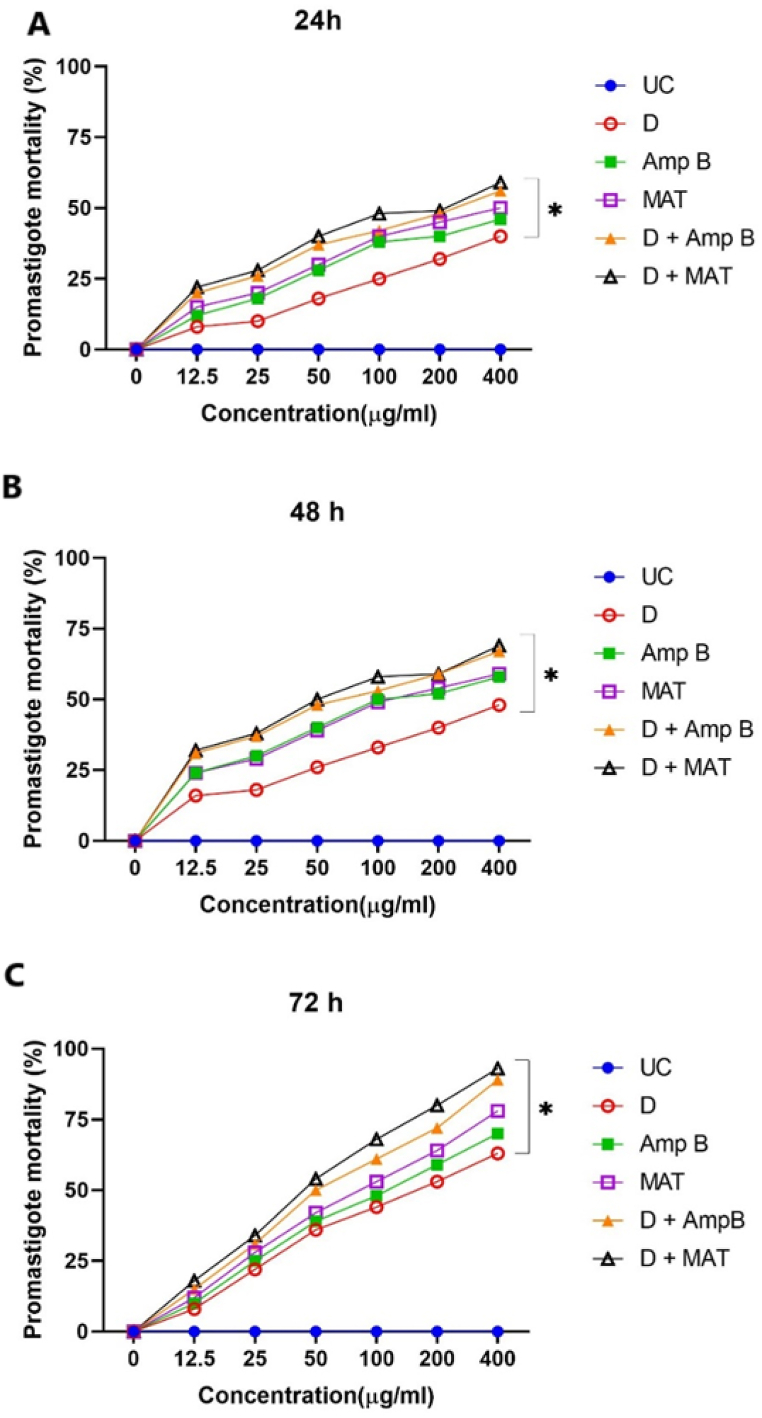
The overall mean mortality rates of *L. major* promastigotes at varying concentrations of D, meglumine antimoniate (MAT), Amphotericin B (AmpB), and their combinations after 24 (a), 48 (b), and 72 (c) hours show a significant difference compared to the untreated control group (UC) (∗P < 0.001).

Fig. 2 illustrates the average mortality rate of promastigotes at different concentrations of D, MAT, and AmpB, both individually and in combination. All treatment modalities exhibited significant anti-leishmanial activity at different concentrations after 24, 48 and 72 h of incubation, compared to the untreated control group (*P* < 0.001).

### 3.2 Antileishmanial activity against *L. major* amastigotes

**Fig. 3:**
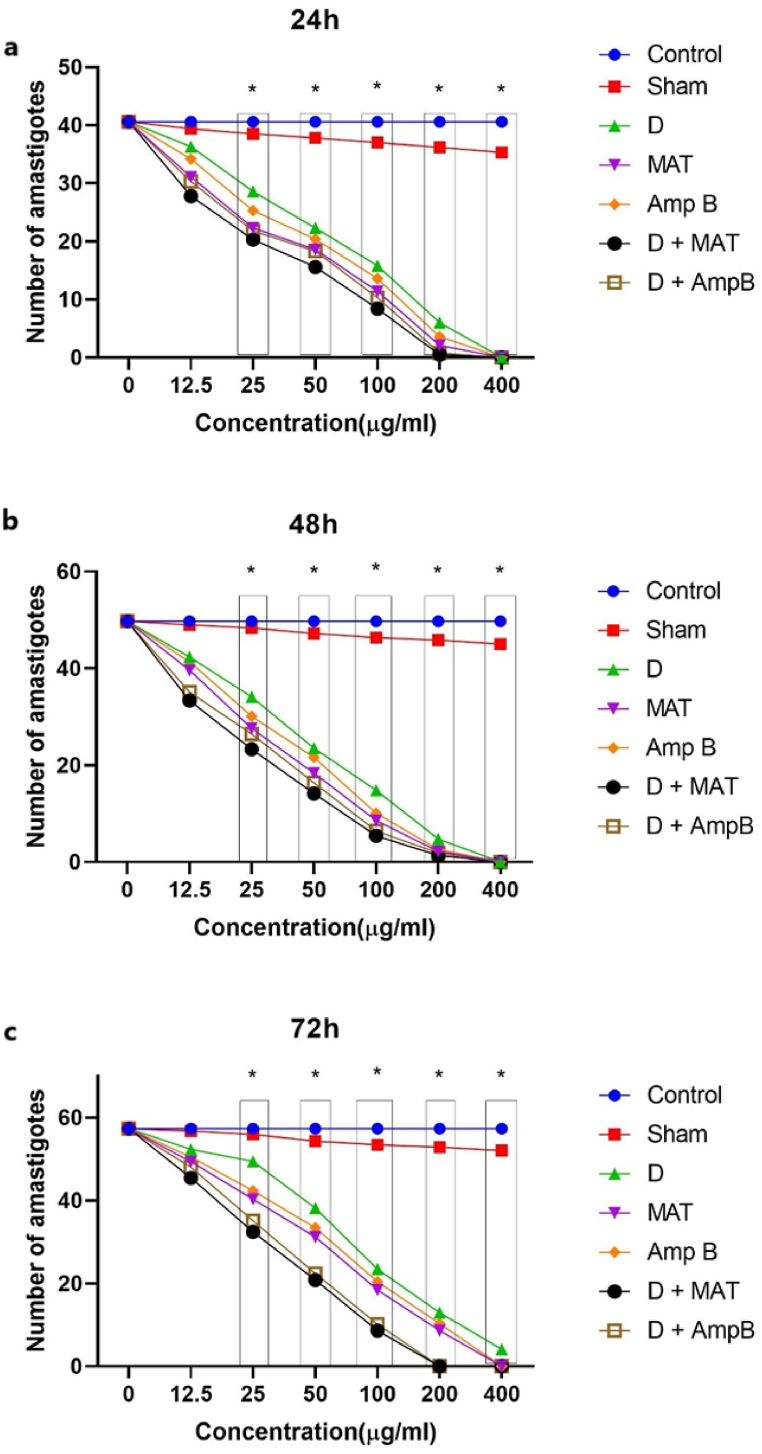
Comparison of the effects of varying concentrations of D, meglumine antimoniate (MAT), and Amphotericin B (Amp B), both individually and in combination, on the average number of intramacrophage amastigotes at 24 (a), 48 (b), and 72 (c) hours, compared to the untreated control group (UC).

Table 2 and Fig. 3 indicate that different concentrations of D, MAT, and AmpB, both alone and in combination, resulted in a significant decrease in the total number of intramacrophage amastigotes compared to the untreated control groups at 24, 48, and 72 h (P < 0.001). All treatments significantly reduced the number of amastigotes at higher concentrations (e.g., 200 μg/mL and 400 μg/mL) and with longer incubation periods, with statistical significance (*P* < 0.001) observed for most concentrations and time points. All tested substances effectively decreased amastigote numbers, indicating their potential effectiveness in leishmaniasis treatment.

**Table 2:** Comparison of the effects of varying concentrations of D, meglumine antimoniate (MAT), and Amphotericin B (Amp B), both individually and in combination, on the average number of intramacrophage amastigotes at 24, 48, and 72 h, relative to the untreated control group (UC).

Time	Concentration (μg/mL)	Sham		D		MAT		Amp B		D + MAT		D + AmpB	
		Mean ± SD	P value	Mean ± SD	P value	Mean ± SD	P value	Mean ± SD	P value	Mean ± SD	P-value	Mean ± SD	P value
24 h	0.0	40.6 ± 4.3	NR	40.6 ± 4.3	NR	40.6 ± 4.3	NR	40.6 ± 4.3	NR	40.6 ± 4.3	NR	40.6 ± 4.3	NR
	12.5	39.4 ± 0.3	P > 0.05	36.3 ± 6.7	P > 0.05	31.1 ± 4.2	P > 0.05	34.2 ± 3.6	P > 0.05	27.8 ± 4.8	P > 0.05	30.3 ± 4.0	P > 0.05
	25	38.5 ± 0.4	P > 0.05	28.6 ± 2.3	P < 0.001	22.3 ± 3.1	P < 0.001	25.3 ± 4.2	P < 0.001	20.3 ± 2.8	P < 0.001	21.8 ± 1.9	P < 0.001
	50	37.8 ± 0.2	P > 0.05	22.3 ± 1.1	P < 0.001	18.6 ± 2.5	P < 0.001	20.4 ± 1.8	P < 0.001	15.6 ± 1.6	P < 0.001	18.3 ± 1.0	P < 0.001
	100	37.0 ± 0.5	P > 0.05	15.8 ± 0.6	P < 0.001	11.4 ± 2.2	P < 0.001	13.6 ± 2.6	P < 0.001	8.4 ± 0.3	P < 0.001	10.3 ± 0.8	P < 0.001
	200	36.2 ± 0.7	P > 0.05	6.0 ± 0.6	P < 0.001	2.1 ± 0.1	P < 0.001	3.6 ± 0.4	P < 0.001	0.5 ± 0.0	P < 0.001	0.8 ± 0.0	P < 0.001
	400	35.3 ± 0.8	P > 0.05	0.0 ± 0.0	P < 0.001	0.0 ± 0.0	P < 0.001	0.0 ± 0.0	P < 0.001	0.0 ± 0.0	P < 0.001	0.0 ± 0.0	P < 0.001
48 h	0.0	49.8 ± 6.6	NR	49.8 ± 6.6	NR	49.8 ± 6.6	NR	49.8 ± 6.6	NR	49.8 ± 6.6	NR	49.8 ± 6.6	NR
	12.5	49.0 ± 2.1	P > 0.05	42.4 ± 8.1	P > 0.05	39.6 ± 1.1	P > 0.05	41.4 ± 3.0	P > 0.05	33.4 ± 2.1	P > 0.05	35.1 ± 2.0	P > 0.05
	25	48.4 ± 1.0	P > 0.05	34.1 ± 4.0	P < 0.001	27.6 ± 1.8	P < 0.001	30.1 ± 2.2	P < 0.001	23.3 ± 2.1	P < 0.001	26.4 ± 1.9	P < 0.001
	50	47.2 ± 2.5	P > 0.05	23.5 ± 1.6	P < 0.001	18.4 ± 2.0	P < 0.001	21.6 ± 2.4	P < 0.001	14.2 ± 3.0	P < 0.001	16.4 ± 1.2	P < 0.001
	100	46.4 ± 1.1	P > 0.05	14.8 ± 0.6	P < 0.001	8.6 ± 0.9	P < 0.001	10.1 ± 1.9	P < 0.001	5.4 ± 0.4	P < 0.001	6.4 ± 0.6	P < 0.001
	200	45.9 ± 1.7	P > 0.05	4.8 ± 0.5	P < 0.001	2.2 ± 0.4	P < 0.001	2.6 ± 0.3	P < 0.001	1.4 ± 0.1	P < 0.001	1.9 ± 0.3	P < 0.001
	400	45.0 ± 0.9	P > 0.05	0.0 ± 0.0	P < 0.001	0.0 ± 0.0	P < 0.001	0.0 ± 0.0	P < 0.001	0.0 ± 0.0	P < 0.001	0.0 ± 0.0	P < 0.001
72 h	0.0	57.3 ± 8.3	NR	57.3 ± 8.3	NR	57.3 ± 8.3	NR	57.3 ± 8.3	NR	57.3 ± 8.3	NR	57.3 ± 8.3	NR
	12.5	56.7 ± 3.1	P > 0.05	52.3 ± 6.7	P > 0.05	49.3 ± 5.7	P > 0.05	50.4 ± 4.8	P > 0.05	45.4 ± 5.0	P > 0.05	48.4 ± 5.6	P > 0.05
	25	55.9 ± 2.5	P > 0.05	49.4 ± 5.1	P < 0.001	40.3 ± 6.4	P < 0.001	42.3 ± 5.2	P < 0.001	32.4 ± 4.4	P < 0.001	35.1 ± 4.1	P < 0.001
	50	54.3 ± 3.1	P > 0.05	38.1 ± 4.0	P < 0.001	31.2 ± 4.1	P < 0.001	33.4 ± 3.0	P < 0.001	20.8 ± 4.1	P < 0.001	22.4 ± 3.4	P < 0.001
	100	53.4 ± 1.1	P > 0.05	23.4 ± 3.8	P < 0.001	18.4 ± 2.5	P < 0.001	20.4 ± 2.4	P < 0.001	8.6 ± 0.8	P < 0.001	10.1 ± 0.5	P < 0.001
	200	52.8 ± 1.3	P > 0.05	12.9 ± 1.4	P < 0.001	8.6 ± 0.2	P < 0.001	10.3 ± 0.8	P < 0.001	0.0 ± 0.0	P < 0.001	0.0 ± 0.0	P < 0.001
	400	52.0 ± 0.9	P > 0.05	4.1 ± 0.2	P < 0.001	0.0 ± 0.0	P < 0.001	0.0 ± 0.0	P < 0.001	0.0 ± 0.0	P < 0.001	0.0 ± 0.0	P < 0.001

### 3.3 The toxicity on macrophages

**Fig. 4:**
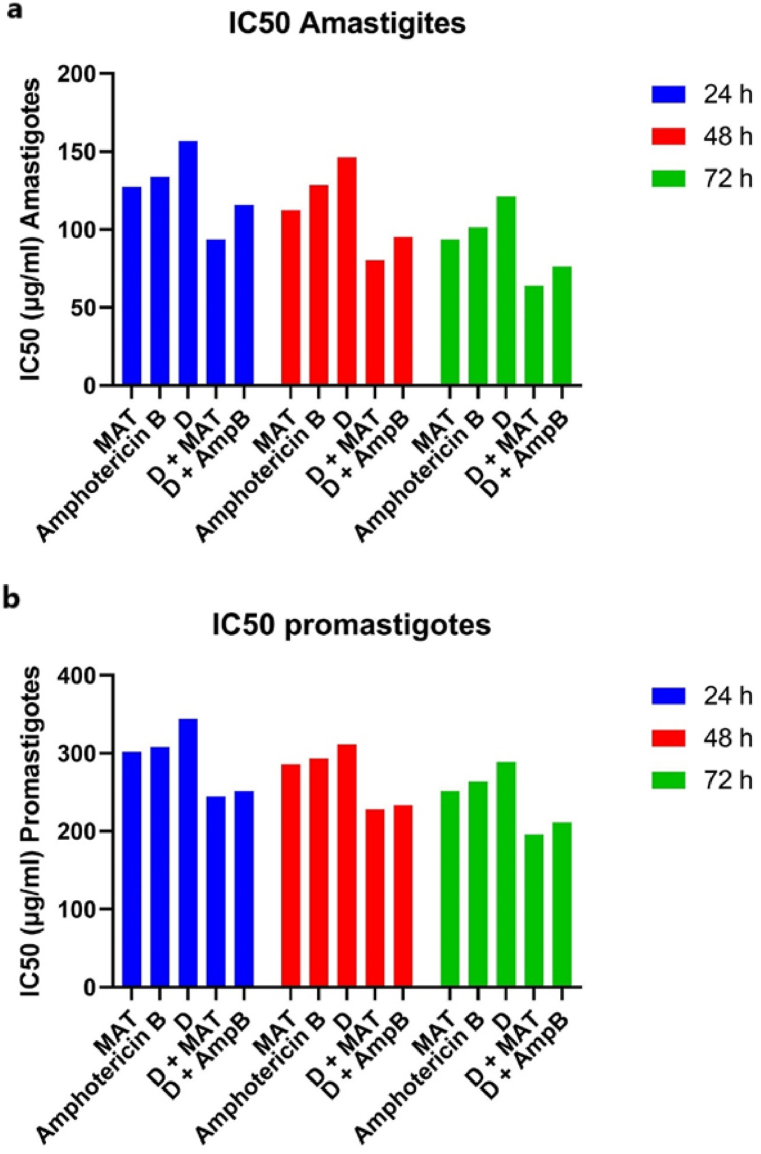
*In vitro* anti-leishmanial (a) and cytotoxic activities (b) of D, D + MAT, D + AmpB, MAT, Amphotericin B in 24 h, 48 h and 72 h compare D group.

Various concentrations of D, MAT, AmpB, and their combinations were evaluated on the macrophage cell line (Table 3 and Fig. 4). The selectivity index (SI), which assesses the cytotoxicity of each treatment, remained within a safe range (Table 3 and Fig. 4). Furthermore, the combination of the two drugs demonstrated greater safety compared to each drug administered alone.

**Table 3:** *In vitro* anti-leishmanial and cytotoxic activities of D, D + MAT, D + AmpB, MAT, Amphotericin B in 24 h, 48 h and 72 h compare D group.

Drug	Amastigote (24 h)			Promastigote (24 h)			Macrophage (24 h)	SI= CC_50_ Macrophages/IC_50_ Amastigotes
	IC_50_ ± SD	*P*-value Compare MAT	*P*-value Compare Amp B	IC_50_ ± SD	*P*-value Compare MAT	*P*-value Compare Amp B	CC_50_	(Selectivity Index)
MAT	127.3 ± 14.9	NR	P < 0.01	301.8 ± 24.3	NR	P < 0.01	780.3 ± 38.2	6.12
Amphotericin B	133.6 ± 18.6	P < 0.01	NR	308 ± 22.0	P < 0.01	NR	792.3 ± 28.6	5.93
D	156.8 ± 18.0	P < 0.001	P < 0.001	344.3 ± 21.3	P < 0.001	P < 0.001	788.6 ± 39.1	5.03
D + MAT	93.6 ± 6.4	P < 0.001	P < 0.001	244.8 ± 19.7	P < 0.001	P < 0.001	759.6 ± 23.7	8.11
D + AmpB	115.9 ± 11.2	P < 0.001	P < 0.001	251.6 ± 14.5	P < 0.001	P < 0.001	763.2 ± 20.8	6.58
**Drug**	**Amastigote (48h)**			**Promastigote (48h)**			**Macrophage (48h)**	**SI** **=** **CC**_**50**_**Macrophages/IC**_**50**_**Amastigotes**
	**IC**_**50**_ ± **SD**	***P*-value** **Compare MAT**	***P*-value** **Compare Amp B**	**IC**_**50**_ ± **SD**	***P*-value** **Compare MAT**	***P*-value** **Compare Amp B**	**CC**_**50**_	**(Selectivity Index)**
MAT	112.6 ± 9.5	NR	P < 0.01	286.2 ± 17.3	NR	P < 0.01	758.3 ± 25.1	6.73
Amphotericin B	128.6 ± 14.2	P < 0.01	NR	293.6 ± 15.3	P < 0.01	NR	772.3 ± 24.6	5.61
D	146.3 ± 11.8	P < 0.001	P < 0.001	311.6 ± 20.6	P < 0.001	P < 0.001	768.3 ± 30.1	5.25
D + MAT	80.6 ± 8.1	P < 0.001	P < 0.001	228.0 ± 28.3	P < 0.001	P < 0.001	735.3 ± 29.4	9.12
D + AmpB	95.3 ± 6.3	P < 0.001	P < 0.001	233.8 ± 16.4	P < 0.001	P < 0.001	744.3 ± 22.2	7.57
**Drug**	**Amastigote (72h)**			**Promastigote (72h)**			**Macrophage (72h)**	**SI** = **CC**_**50**_**Macrophages/IC**_**50**_**Amastigotes**
	**IC**_**50**_ ± **SD**	***P*-value** **Compare MAT**	***P*-value** **Compare Amp B**	**IC**_**50**_ ± **SD**	***P*-value** **Compare MAT**	***P*-value** **Compare Amp B**	**CC**_**50**_	**(Selectivity Index)**
MAT	93.6 ± 15.2	NR	P < 0.01	251.6 ± 28.4	NR	P < 0.01	744.9 ± 25.0	7.95
Amphotericin B	101.2 ± 18.3	P < 0.01	NR	263.8 ± 20.7	P < 0.01	NR	748.9 ± 18.6	7.40
D	121.3 ± 22.8	P < 0.001	P < 0.001	288.4 ± 19.4	P < 0.001	P < 0.001	751.3 ± 32.7	6.19
D + MAT	63.9 ± 8.4	P < 0.001	P < 0.001	196.1 ± 10.2	P < 0.001	P < 0.001	720.6 ± 38.7	11.27
D + AmpB	76.3 ± 5.7	P < 0.001	P < 0.001	211.3 ± 24.9	P < 0.001	P < 0.001	739.6 ± 30.7	9.69

NR: Not related.

### 3.4 Antioxidant activity

**Fig. 5:**
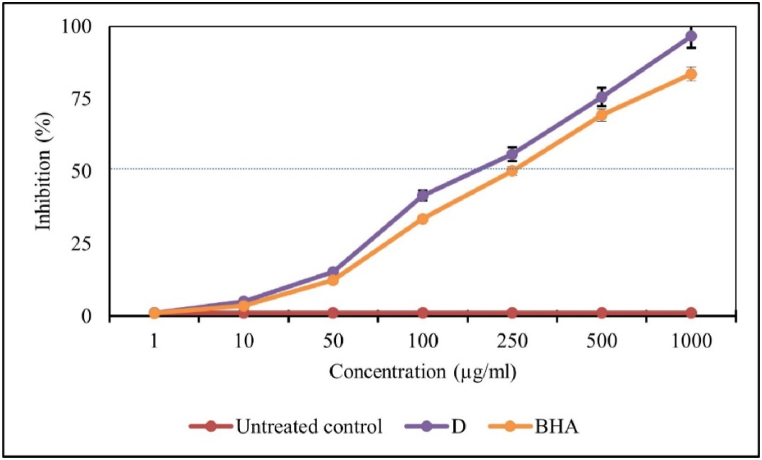
The scavenging activity of different concentrations of D and butylated hydroxyanisole (BHA), used as a positive control, on 1,1-diphenyl-2-picrylhydrazyl (DPPH) free radicals was compared to the untreated control. Bars represent the mean ± standard deviation from triplicate experiments.

Fig. 5 demonstrates the antioxidant activity of D and BHA, used as a positive control, measured by the DPPH method. The results showed high rate of radical-scavenging activity for both the D and BHA, following a dose-response pattern. The IC50 value for D was calculated to be 226.3 μg/mL, which is significantly lower than that for BHA, which was 249.3 μg/mL (P < 0.001). Furthermore, D demonstrates greater effectiveness in reducing the stable free radical DPPH to its yellow-colored form compared to the untreated control.

### 3.5 Th1 and Th2 related cytokines

**Fig. 6:**
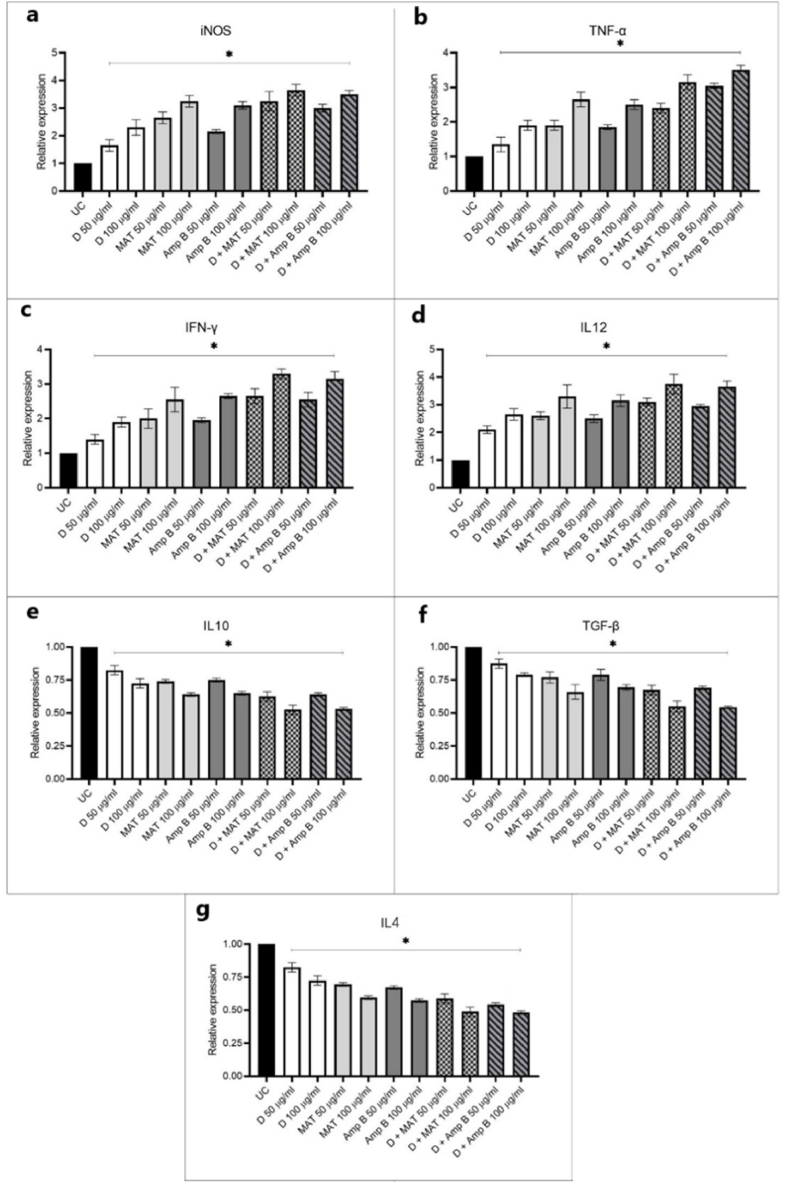
The gene expression profiles and protein products in macrophages related to Th1 and Th2 cytokines were analyzed, focusing on iNOS (a) and Th1-related cytokines such as TNF-α (b), IFN-γ (c), and IL-12 (d), as well as Th2-related cytokines including IL-10 (e), TGF-β (f), and IL-4 (g). These were evaluated at different concentrations of D, meglumine antimoniate (MAT), Amphotericin B (AmpB), both individually and in combination, compared to the control group. Bars represent standard deviation (∗P < 0.001).

Fig. 6 illustrates the expression levels of Th1 and Th2 cytokine genes. The results indicate that the treated groups exhibited significantly higher gene expression of Th1 cytokines compared to the control group (P < 0.001). Specifically, Th1 cytokines, which include TNF-α (Tumor Necrosis Factor-alpha), IFN-γ (Interferon-gamma), and IL-12 (Interleukin-12), were markedly elevated in response to treatment. TNF-α is crucial for macrophage activation and inducing an inflammatory response. IFN-γ is important for enhancing the antigen presentation and stimulating Th1 responses. IL-12 plays a key role in promoting Th1 differentiation and inducing IFN-γ production.

### 3.6 Apoptotic profiles

**Fig. 7:**
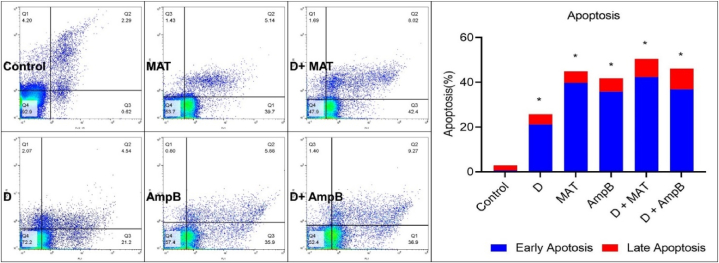
Flow cytometry analysis was conducted on *L. major* amastigotes exposed to different concentrations of 100 μg/mL of D, MAT, AmpB, and their combination, compared to the untreated control after 72 h of incubation. Bars represent the mean ± standard deviation of viability rates (∗*P* < 0.001).

Intra-macrophage amastigotes treated with 100 μg/mL of D, MAT, AmpB, and their combination exhibited significant induction of apoptosis. All three treatment regimens, administered at IC₅₀ concentrations, showed a marked difference compared to the negative control group (P < 0.001) (Fig. 7).

### 3.7 Observation of morphological changes with D

**Fig. 8:**
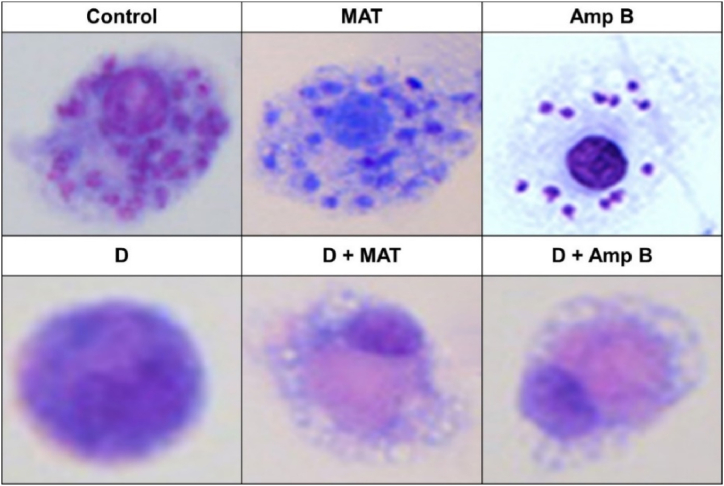
Cellular Morphology by Giemsa Staining ( × 1000) after treatment with various sample. The staining highlights the intracellular amastigotes within the macrophages, allowing for the assessment of morphological changes induced by the treatments.

Fig. 8 displays the cellular morphology of *L. major* amastigotes inside macrophages, as observed through Giemsa staining ( × 1000) after treatment with the control, D, MAT, AmpB, and their combinations.

## 4 Discussion

Leishmaniasis presents a significant challenge as one of the prevalent protozoal parasitic diseases, affecting over one million individuals annually in tropical and subtropical regions, with a population at risk 350 million [24]. Despite ongoing efforts to identify effective treatments, a superior drug remains elusive due to various factors such as diverse side effects and the development of resistance by parasites to chemotherapy [25]. Extensive research has focused on exploring natural products, particularly medicinal plants, to find potential treatment options [25,26]. The study investigated the *in vitro* efficacy of *D. lindbergii* against *L. major*, focusing on its ability to inhibit growth, induce apoptosis, and modulate cytokine expression. The results revealed promising anti-leishmanial activity of fraction D, supported by its significant antioxidant potential and selective cytotoxicity.

The assessment of anti-*Leishmania* activity involved determining the IC50 values for various fractions (F1, F2, F3, D), as well as the reference drugs meglumine antimoniate (MAT) and Amphotericin B (AmpB), both individually and in combination, against amastigotes, promastigotes, and macrophages at 24, 48, and 72 h. Notably, the combination of D + MAT exhibited the most robust anti-*Leishmania* activity against amastigotes after 24 h and demonstrated the highest activity against promastigotes. Additionally, D + MAT showed significant activity against macrophages, indicating a favorable selectivity index (SI). The findings revealed time-dependent variations in the anti-*Leishmania* activity, with effectiveness generally improving over the 72-h period, surpassing the results obtained at 24 and 48 h. Previous studies have reported that several medicinal plants possess moderate to high anti-leishmanial activity [22,27].

Other authors have identified certain plants that could serve as adjuvants in vaccines against *Leishmania* spp [12,13]. In terms of anti-leishmanial activity, D demonstrated significant efficacy in inhibiting the growth of both promastigotes and intramacrophage amastigotes of *L. major*. This activity was evident across various concentrations and time points, indicating a dose-dependent effect. The observed cytotoxicity of D against intracellular amastigotes is particularly noteworthy, given the challenge posed by intracellular parasites in leishmaniasis treatment. Combination therapy is often used to enhance efficacy and reduce drug resistance. In this study, the combination of D with conventional anti-leishmanial drugs, meglumine antimoniate (MAT), and Amphotericin B (AmpB), exhibited synergistic effects. This mixture not only enhanced anti-leishmanial activity but also demonstrated a safer cytotoxic profile compared to the individual drugs, as indicated by the Selectivity Index (SI).

Regarding cellular morphology, Giemsa staining and light microscopy revealed significant alterations in the morphology with treated fraction D. These changes included cell shrinkage, cytoplasmic and condensation, particularly evident after 24 h of treatment. In contrast, the control group exhibited no significant morphological changes. Flow cytometry analysis with annexin-V FLUOS staining confirmed that fraction D induced apoptosis in promastigotes. After 72 h of incubation, the percentage of promastigotes in early and late apoptosis varied over time in the experimental group, consistent with findings from Elamin et al. (2021) [18], who reported curcumin's anti-proliferative and apoptosis-inducing activities on *L. major*. Their study demonstrated that curcumin led to a maximum accumulation of S-phase cells at about 33 % after 16 h of incubation, compared to 14 % in the control group.

Additionally, fraction D showed variable effects on cytokine levels, with a more pronounced impact on IL-10, followed by IL-4 and TGF-β. Treatment with fraction D and Amphotericin B led to a reduction in promastigote mobility over time, with Amphotericin B inducing cell death after 48 and 72 h. The study also assessed the modulation of the immune response by fraction D, finding that it significantly upregulated the expression of Th1-related cytokines (TNF-α, IFN-γ, IL-12). This indicates a shift towards a protective immune response against leishmaniasis. The modulation of cytokine expression aligns with the observed anti-leishmanial activity of fraction D, suggesting its role in both direct parasite inhibition and enhancement of the immune response.

The antioxidant activity of various concentrations of fraction D and BHA (butylated hydroxyanisole) was assessed using the DPPH radical-scavenging method. The results demonstrated a strong radical-scavenging activity for both D and BHA, following a dose-dependent pattern. Statistical analysis determined the IC50 value for D to be 226.3 μg/mL, which is significantly lower than that of BHA at 249.3 μg/mL (P < 0.001). This suggests that fraction D exhibits greater efficiency in scavenging DPPH radicals compared to BHA.

The high radical-scavenging activity of D underscores its potential as an effective antioxidant agent. The observed dose-response relationship highlights D's ability to decrease stable free radicals in a concentration-dependent manner. Given the role of oxidative stress in the pathogenesis of leishmaniasis, the antioxidant properties of fraction D could contribute to its therapeutic efficacy. The superior radical-scavenging activity of D compared to BHA further supports its potential for use in antioxidant and anti-leishmanial applications.

In the context of medicinal plants with potential therapeutic effects, it is noteworthy that both *D. kotschyi* and *D. lindbergii* belong to the same family, Lamiaceae. According to Kosari and Khamesipour, (2022) [28], inducing programmed cell death (apoptosis) in *L. major* promastigotes using medicinal plants like *D. kotschyi* presents a promising alternative to conventional treatments, potentially offering effective parasite elimination. Shaabani et al. (2020) [29] further supported this notion in their research on human cancer cells, where they found that the essential oil of *D. kotschyi* triggered apoptosis in human glioblastoma U87 cells in a dose-dependent manner. Their study noted a significant increase in cells in the sub-G1 phase compared to controls, indicating apoptosis. Additionally, the proliferation of U87 cells was affected, and the apoptotic effects were linked to the influence of *D. kotschyi* extracts on reactive oxygen species (ROS), which are critical in regulating cell growth, inflammatory responses, and cell death [29,30].

The induction of apoptosis in intramacrophage amastigotes by fraction D, as observed in this study, underscores its potential as a therapeutic agent against *L. major*. Apoptosis is particularly beneficial in leishmaniasis treatment as it promotes parasite clearance while minimizing damage to host tissues. The increase in apoptosis rates following D treatment highlights its efficacy and potential for further development as an effective anti-leishmanial therapy.

Drawing on existing literature, the findings from this study align with established research on the anti-leishmanial properties of medicinal plants. Fraction D of *D. lindbergii* demonstrated notable anti-*Leishmania* activity, particularly against *L. major* amastigotes and promastigotes. This result is consistent with prior studies that highlight the efficacy of plant extracts in combating parasitic infections. The enhancement of anti-leishmanial effects observed with prolonged exposure to fraction D aligns with research suggesting that extended treatment duration can improve therapeutic outcomes in anti-parasitic therapies.

Additionally, the induction of apoptosis in *L. major* promastigotes by fraction D supports its anti-parasitic potential. Apoptosis is a well-recognized mechanism for targeting parasitic infections, and its role in leishmaniasis treatment has been previously documented. The observed changes in cellular morphology, such as reduced mobility, cell shrinkage, and other apoptotic signs, further validate the effectiveness of fraction D in combating *L. major*. These indicators of successful anti-parasitic activity emphasize the potential of *D. lindbergii* as a valuable therapeutic agent for leishmaniasis.

Overall, this study highlights the potential of *D. lindbergii* as an anti-*Leishmania* agent. The study demonstrates the significant *in vitro* anti-leishmanial activity of D. lindbergii, attributed to specific chemical compounds isolated. The time-dependent enhancement of activity, induction of apoptosis, and observed morphological changes in *L. major* align with existing literature on effective anti-leishmanial treatments. The significant findings from the 100 % methanolic fraction of *D. lindbergii* suggest it could be a valuable candidate for developing improved traditional drugs for leishmaniasis. Further research and clinical trials are essential to fully assess and harness the therapeutic potential of this medicinal plant and also research into other *Leishmania* species to validate these results.

## Ethics statement

Not applicable.

## Data availability statement

Not applicable.

## Funding

Not applicable.

## Consent for publication

Not applicable.

## CRediT authorship contribution statement

**Faham Khamesipour:** Writing – review & editing, Writing – original draft, Methodology, Investigation, Conceptualization. **Ali Khamesipour:** Writing – review & editing, Visualization, Validation, Supervision, Conceptualization. **Seyed Hossein Hejazi:** Writing – review & editing, Validation, Methodology. **Mustafa Ghanadian:** Writing – review & editing, Validation, Methodology.

## Declaration of competing interest

The authors declare that they have no known competing financial interests or personal relationships that could have appeared to influence the work reported in this paper.

## Abbreviations

*D. lindbergii*: *Dracocephalum lindbergii*
*L. major*: *Leishmania major*
DMSO: Dimethyl sulfoxide
HEPES: 4-(2-hydroxyethyl)-1-piperazine-ethanesulfonic acid
MTT: 3-(4, 5-dimethylthiazol-2-yl)-2, 5-diphenyltetrazolium bromide
PBS: phosphate-buffered saline
FBS: fetal bovine serum
DPPH: 2,2-diphenyl-1-picrylhydrazyl
RI: Retention indices
Rt: Retention time
TIC: Total ion count
THP-1: A macrophage cell line
